# Tibiotarsal mechanical behaviour is mainly a function of bone structure and not mineral density in female adolescent chickens

**DOI:** 10.3389/fbioe.2026.1812701

**Published:** 2026-09-28

**Authors:** Isabela Vitienes, Catherine Julien, Ana K. Rentsch, Russell P. Main, Tina M. Widowski, Sara Checa, Bettina M. Willie

**Affiliations:** 1 Department of Biological and Biomedical Engineering, McGill University, Montreal, QC, Canada; 2 Research Centre, Shriners Hospital for Children-Canada, Montreal, QC, Canada; 3 Department of Operative, Preventive and Pediatric Dentistry, Charité – Universitätsmedizin, Berlin, Germany; 4 Faculty of Dental Medicine and Oral Health Science, McGill University, Montreal, QC, Canada; 5 Department of Animal Bioscience, University of Guelph, Guelph, ON, Canada; 6 Weldon School of Biomedical Engineering, Purdue University, West Lafayette, IN, United States; 7 Department of Basic Medical Sciences, Purdue University, Purdue, IN, United States; 8 Julius Wolff Institute, Charité-Universitätsmedizin Berlin, Berlin, Germany; 9 Institute of Biomechanics, Hamburg University of Technology (TUHH), Hamburg, Germany; 10 College of Veterinary Medicine, Utah State University, Logan, UT, United States

**Keywords:** bone adaptation, bone fragility, bone mechanical behaviour, bone structure, egg-laying hens

## Abstract

**Introduction:**

Bone fragility and disuse osteoporosis in commercial egg-laying chickens is a major welfare concern. Efforts to address this have focused on eliciting increases in bone mass during growth by raising chickens in more spacious and complex housing systems that motivate increased physical activity. However, it remains unclear how these bone adaptations influence *in vivo* bone mechanical behaviour in chickens, namely, whole-bone stiffness and strength.

**Methods:**

To investigate this, we sampled female chickens of two genetic strains (Lohmann Brown: brown-feathered, Lohmann LSL Lite: white-feathered) with different loading histories (sedentary: housed in cages with no opportunity for dynamic load bearing activity; active: raised in one of two styles of aviary housing systems requiring locomotion including jumping and other aerial transitions) just prior to the onset of sexual maturity at 16 weeks of age. We imaged their whole right tibiotarsi (*ex vivo*) using microCT and for the first time developed finite element models of the chicken tibiotarsi under controlled *in vivo* axial compressive loading on a limited sample size (n = 1, N = 6), which we validated with *in vivo* strain gauge measurements. Each bone was modelled with both homogeneous and heterogeneous material properties to assess the effects of tissue mineral density and bone structure on the mechanical behaviour. We also quantified a variety of structural measures along the bone length and determined their correlation to simulated mechanical behaviour.

**Results:**

We found that consideration of volumetric tissue mineralization heterogeneity in our FE models had negligible influence on the modelled mechanical behaviour, and thus further analyses used only homogeneous FEMs. We identified bone area, total area, bone area ratio, bone thickness, eccentricity, normalized outer perimeter, and inner-to-outer perimeter ratio to be significantly correlated to decreased modelled stress. Increased activity levels was associated with lower modelled stresses in brown-, but not white-feathered chickens, and this pattern could be explained by differences in bone area, total area, and inner-to-outer perimeter ratio.

**Conclusion:**

These results suggest that, similar to what has been shown in humans, bone structure and not just mineral density is a critical determinant of mechanical behaviour and therefore fracture risk. We identify structural outcomes that can be candidates for genetic selection, targeting improved bone strength, a subset of which may adapt to loading in brown-feathered chickens. We also present a novel (in the poultry science field) approach to assess the relationship between bone structure and mechanical behaviour, which can be further used to inform genetic selection and breeding for improved bone strength and fracture resistance.

## 1 Introduction

Commercial laying hens suffer from osteoporosis and high fracture incidence (Whitehead and Fleming, 2000), which poses a significant welfare concern. Fracture risk is dependent on whole bone stiffness which is a function of 1) the properties of the bone material such as its elastic modulus, and 2) the arrangement of the bone tissue in space (bone structure) (van der Meulen et al., 2001; Burr, 2019). Bone structure itself depends on how much bone there is (bone volume) and architectural features such as whole bone curvature, bone length, cross-sectional thickness, cross-sectional area, among others. Osteoporosis occurs when there is impaired bone material properties, such as low bone mineral density, and/or deteriorated bone structure, thereby decreasing both the bone’s overall stiffness and strength. Despite being often considered a disease with an adult onset, research in humans shows that susceptibility to adult-onset osteoporosis is largely determined by adolescent bone development and peak bone mass achieved (Rosen, 2000). In humans, bone mineral density has historically been the main clinical outcome measure to assess osteoporosis and fracture risk, due to widespread availability and non-invasiveness of DXA scanning. It is only recently that non-invasive methods to image bones in 3D at sufficiently high resolution have become more widely used in clinics. Thanks to these advancements, recent studies have found that high fracture incidence in women with seemingly healthy BMD levels can be attributed to deleterious, weakening alterations in bone structure (Samelson et al., 2019; Mikolajewicz et al., 2020; Sarfati et al., 2024). As in humans, many studies to date seeking to assess the severity of osteoporosis in commercial laying hens and test the effects of interventions have looked at bone mineral density, ash density, and 2D radiography-based measures of bone geomtry as indicators of bone stiffness and strength (Kim et al., 2012; Chen and Kim, 2020; Sallam et al., 2024; 2025). Only recently have studies begun using high-resolution computed tomography (CT) imaging to distinguish cortical and trabecular bone and evaluate their structural properties (Regmi et al., 2017; Chen and Kim, 2020) as indirect measures of resistance to fracture. To date no studies have used this 3D CT data for finite element simulations to predict whole bone mechanical behaviour; this instead has exclusively been assessed by destructive *ex vivo* mechanical testing (e.g., 3-point bending).

Providing increased opportunities for physical activity by housing chickens in more spacious and complex environments has positive effects on bone health related outcomes especially when these opportunities are provided from youth (Fleming et al., 2006; Enneking et al., 2012; Hester et al., 2013; Heerkens et al., 2016; Casey-Trott et al., 2017a; 2017b; Ciarelli et al., 2023). Specifically for the genetic strains and housing systems assessed in this current study, we have previously shown that chickens housed in cages are less active than those housed in aviaries (open-concept non-cage housing systems) (Rentsch et al., 2023). The cages were restrictive, only allowing chickens to stand, sit, or at most take a few steps from one side of the cage to the other (Figure 1). Aviaries instead were substantially more spacious, with varied infrastructure that not only provides opportunity for, but requires that each chicken navigate between tiers to access food, water, and litter (Figure 1). We have also shown that chickens raised in aviaries had wider, longer bones with higher cortical bone thickness, and higher bone mineral content compared to chickens raised in cages (Vitienes et al., 2026). It remains unclear, however, how these adaptations relate to whole-bone stiffness and fracture resistance.

**FIGURE 1:**
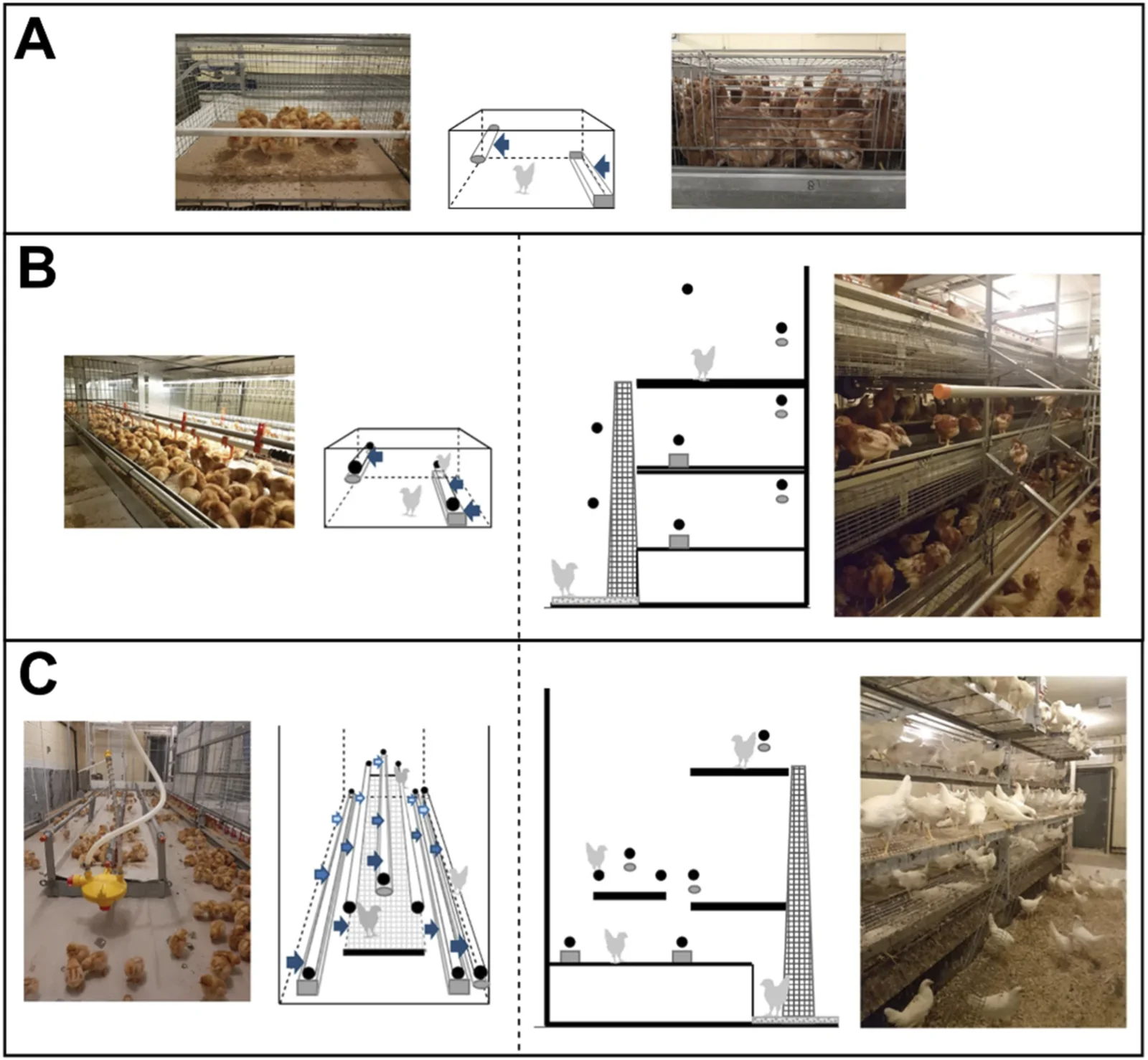
Housing systems. In all illustrations, grey ovals represent drinkers, grey boxes are feed troughs, black circles are perches, and black rectangles are platforms. **(A)** Conventional cage (Conv). The image on the left shows the cage with young (<6 weeks of age) pullets while the image on the right is the same group of pullets at >10 weeks of age. In the center is an illustration of the cage and its components. **(B,C)** Images and illustrations of the low **(B)** and high **(C)** complexity aviaries during (0–6 weeks of age, left of dashed line) and after (6–14 weeks of age, right of dashed line) the brooding phase. Images and illustrations are adapted from Rentsch et al. (2023).

We aimed to address this gap in knowledge by performing finite element analyses modelling physiological controlled *in vivo* axial compressive loading of the tibiotarsus to characterize the mechanical behaviour of entire tibiotarsi and assess the influence of bone mineral density and microstructure on this behaviour, an approach and methodology that has yet to be applied in chicken bones. Moreover, we sought to compare these data between chickens with active or sedentary loading histories, to understand by which forms of adaptations can physical activity improve bone strength. We sampled bones from two genetic strains of chickens that were raised in one of three styles of housing: a conventional cage that allowed for no dynamic load bearing activity (Sedentary), and two types of aviary-style systems allowing for moderate or high levels of physical activity (Active). We hypothesized that 1) both tissue mineral density and bone structure would be important determinants of mechanical behaviour in all bones samples, and 2) active chickens in each genotype would have improved microstructure compared to the sedentary chicken which would coincide with lower FE modelled stresses.

## 2 Materials and methods

We combined *ex vivo* experiments and finite element modelling to examine how adaptations to different loading histories relate to whole-bone mechanical behaviour and fracture resistance. Female chickens of two genetic strains were reared in various housing conditions which imposed either sedentary or active loading histories. Sampled chickens were instrumented with strain gauges and subjected to controlled axial compressive loading, to obtain *in vivo* strain measurements used to validate finite element models of this axial compressive loading. The finite element model geometry was obtained from microCT images, and were used to predict strains and stresses throughout the entirety of each sampled bone. Two versions of the models were created per bone, one with homogeneous bone mineral density, and one with realistic heterogeneous bone mineral density (acquired by calibrating the microCT greyvalues), to assess the effect of bone mineral density heterogeneity on bone mechanical behaviour. Bone structural outcomes were also measured from the microCT images, to assess the relationship between bone structure and mechanical behaviour.

### 2.1 Housing and management

All animal use and procedures were approved by the animal care committees at the University of Guelph (AUP #4127) and McGill University (AUP #8083). Housing and management are briefly described, whereas full details are reported elsewhere (Vitienes et al., 2023). Over a 2-year period, three consecutive flocks of Lohmann Selected Leghorn Lite (white-feathered) and Lohmann Brown Lite (brown-feathered) female chicks (*Gallus gallus domesticus*) were obtained from a commercial hatchery at the age of 1 day old. The chickens were fed *ad libitum* and reared from 1 day of age to 14 weeks at the University of Guelph in either conventional rearing cages (Conv: Ford Dickinson, Mitchell, Ontario, Canada), or one of two styles of commercial aviaries, allowing for either moderate or high levels of dynamic load-bearing activity. Conventional cages are spatially restrictive and do not allow for dynamic load-bearing activity–chickens are only able to stand and sit, thereby experiencing a state of disuse. The two aviaries alternatively are more spacious and contain infrastructure that requires locomotion and jumping to reach food, water, and litter used by chickens for dustbathing and scratching (Figure 1) (Pufall et al., 2021; Rentsch et al., 2023). Activity level measurements for these chickens are reported elsewhere (Rentsch et al., 2023). The different housing led to distinct loading histories: 1) sedentary: conventional cages; 2) active: one of two styles of aviary housing systems. Just prior to the onset of sexual maturity at 16 weeks of age, a single chicken from each experimental group (n = 1/genetic strain (2)/housing system (3), N = 6) were sampled and transported to McGill University for further measurements.

Chickens of each genetic strain were housed separately. Conventional cages were populated with 30 chickens/cage (h: 40 cm, w: 76 cm, d: 66 cm) during weeks 0–6 (167 cm^2^/chicken), and 15 chickens/cage during weeks 6–14 (344 cm^2^/chicken). For each flock, a total of 300 chickens per genetic strain were reared in Low aviaries, whereas the High housing group had 600 chickens per genetic strain per flock. All other conditions (feed, vaccination, lighting, temperature) were held constant across housing groups.

Upon arrival at McGill, the white-feathered and brown-feathered chickens were housed in separate identical rooms consisting only of litter floor (total area of 13.2 m^2^). Chickens were fed *ad libitum* standard age-appropriate crumble diet. A lighting schedule of 10 h of light per day, at 10 lux, was maintained, and rooms were kept at 20 °C. Chickens were allowed to acclimate for at least 72 h prior to the onset of the experiment. Chickens were weighed the morning prior to undergoing surgery for strain gauge attachment.

### 2.2 *In vivo* strain measurements at the tibiotarsal midshaft during axial compressive loading

Sampled chickens underwent strain gauging during controlled *in vivo* axial compressive loading to obtain experimental strain measurements used to validate our finite element predictions of strain during simulated controlled axial compressive loading. Chickens were instrumented with strain gauges at the right tibiotarsus midshaft (Supplementary Figure S1). Strain gauge preparation and surgical procedures to attach strain gauges are reported elsewhere (Vitienes et al., 2023). Briefly, chickens were anesthetized (sedation: 0.5%–1% of isoflurane at 1 L/min O2 flow rate using a mask, maintenance: 2%–4% isoflurane at 1L/min O2 flow rate via intubation) and underwent surgery during which strain gauges were attached to their tibiotarsus midshaft. Sufficient anesthetic depth was asserted via the loss of toe pinch reflex and palpebral reflex. Following a 2–3 h period of recovery from surgery, chickens underwent *in vivo* background strain gauging (results reported in (Vitienes et al., 2023)), after which time the chickens were anesthetized again for strain gauging during controlled loading. The hindlimb was then secured in the loading device (Testbench ElectroForce LM1, Bose, Framingham, United States of America) and the tibiotarsi were subjected to bouts of controlled axial compressive load (−120N) while engendered strains were recorded from a rosette strain gauge (FR-1-11, Tokyo Sokki Kenkyujo Co., Ltd, Tokyo, Japan) attached to the anterior surface of the tibiotarsus midshaft. We applied dynamic compressive loading at a frequency of 4 Hz by a triangular waveform with a 0.1s rest between each cycle, for 216 load cycles. Applied peak loads, measured by a load cell, differed slightly from the target load level (−125 ± 4 N average applied load). A pre-load of −10 N was maintained during the rest phase to hold the limb in place. The target load value was selected as it engenders principal strains ranging from 236 to 502 με at the tibiotarsus anterior midshaft, which are similar in magnitude to what we previously measured during *in vivo* background activities (Vitienes et al., 2023). The loading device was outfitted with custom 3D printed knee and ankle fixtures. Strain signals were measured by connecting the surgically attached strain gauges to a 3 m long shielded cable (326-BSV; Micro-Measurements, United States of America) connected to a bridge amplifier (System 8,000 Scanner; Micro-Measurements, United States of America). Strains were recorded (StrainSmart, Micro-Measurements, United States of America) using an excitation voltage of 3V and sampled at 1 kHz. A total of 4 channels were recorded, corresponding to the three elements of the rosette strain gauge at the anterior surface and input from the loading machine load cell, such that load and strain were recorded simultaneously. Immediately following controlled loading (while anesthetized), chickens were euthanized (placed in CO_2_-filled clear chamber until lack of respiratory activity for 5 min, followed by cervical dislocation; verification of death via eye and toe pinch reflex), and their carcasses frozen for later dissection, after which time the strain gauged tibiotarsi were dissected and stored in 70% ethanol.

### 2.3 Microcomputed tomography of the tibiotarsus

Whole tibiotarsi were imaged at an isotropic voxel size of 40.5 μm (in air, Skyscan 1,276, Bruker, Massachusetts, United States of America; 80 kV, 200 μA, 125 m exposure time, 0.8° rotation step, Aluminum 0.5 mm filter, 180° scan, no frame averaging). Images were reconstructed with a dynamic range of 0–0.055 cm^-1^ and corrections for misalignment were applied when needed. To limit the possible effects of beam hardening, we implemented aluminum beam filtration during image acquisition and a correction algorithm provided by the manufacturer (NRecon, Bruker, Massachusetts, United States of America) during scan reconstruction (Meganck et al., 2009). MicroCT images were calibrated to units of gHA/cm^3^ with calibration equations derived by imaging calibration phantoms (0.25 and 0.75 gHA/cm^3^), measuring the average attenuation coefficient of each phantom ( ${\bar{\mu }}_{0.25}$ ; ${\bar{\mu }}_{0.75}$ ), and solving the system of equations assuming a linear relationship between bone density ( ρ ) and x-ray attenuation coefficient ( $\left.\mu \right)$ to derive calibration equation constants *a* and *b*:

$$\left\{\begin{array}{c} 0.25\frac{gHA}{{cm}^{3}}=a\;{\bar{\mu }}_{0.25}+b\\ 0.75\frac{gHA}{{cm}^{3}}=a\;{\bar{\mu }}_{0.75}+b \end{array}\right.$$

Bones were then binarized using a global threshold of 0.22 gHA/cm^3^ to obtain masks of the bone tissue, excluding background and soft tissue. The fibula was segmented and excluded from all analyses given a lack of bony connection to the tibiotarsus, implying it does not bear load.

### 2.4 Finite element model prediction of load-induced strain and stress across the entire tibiotarsus

We employed finite element modelling to predict the mechanical strain and stress environment produced under physiological axial compressive loading conditions. Briefly, whole-bone geometry was obtained from microCT-derived tetrahedral meshes, with material properties assigned to each element. Boundary conditions–contact surfaces, load magnitude, and load direction–were defined to replicate *in vivo* axial compressive loading. From these inputs, the model solved for the resulting deformation of the bone, from which principal strains (tensile and compressive) and von Mises stress was derived for each tetrahedral element.

#### 2.4.1 Whole bone geometry

Binarized whole bone microCT images were converted to tetrahedral meshes using Amira 3D software (Amira 3D 2022, Thermo Fisher Scientific, Massachusetts, United States of America) (Table 1) for finite element modelling (Abaqus, Dassault Systems Simulia, Johnston, RI, United States of America).

**TABLE 1:** FEA parameters and validation. Max (abs) principal strain probed in FE models at the location of gauge attachment and principal tensile strain measured experimentally by strain gauging at the anterior midshaft surface during *in vivo* controlled loading.

​	​	​	​	​	​	Heterogeneous		Homogeneous		
Genetic strain	Housing	Bone length [mm]	# FE elements [x 106]	Element characteristic length [mm]	Experimental strain [με]	FE strain [με]	Δ(exp - FE)	FE strain [με]	Δ(exp - FE)	
B	Conv	118	2.49	0.328	340	135	205	213	127	
B	Low	117	3.62	0.306	390	311	79	393	−3	
B	High	124	2.61	0.344	502	437	65	541	−39	
W	Conv	122	2.81	0.274	478	272	206	459	19	
W	Low	120	2.90	0.288	236	152	84	245	−9	
W	High	116	3.66	0.277	268	198	70	209	59	
​	​	120 ± 3	2.88 ± 0.51	0.303 ± 0.028	369 ± 108	251 ± 114	118 ± 68	343 ± 141	26 ± 59	Mean ± sd

#### 2.4.2 FEM boundary conditions

Boundary conditions replicated the controlled loading experiment (Figure 2A). Two reference points were placed at each end of the bone: at the medial and lateral condyle centers (distal) and at the centers of the contact points of the two femoral condyles (proximal). The midpoints of each pair of reference points were then used to define the bone’s long axis (as the line joining these two midpoints). Model contact surfaces were manually selected as the flexed tibiofemoral contact surface (proximal) and the contact surface between the distal tibiotarsus and the ankle fixture (distal) (Figure 2B). A load of 120 N was applied at a point on the bone’s long axis translated 20 mm distally from the distal contact surface. This load level was selected as it engenders midshaft strains (370 με, mean across the 6 FE bones) comparable in magnitude to those engendered during *in vivo* locomotion in both genetic strains (Vitienes et al., 2023). The force vector was oriented at a 10° angle from the bone’s axis in the posterior direction (Figure 2A). During our model development, we also tested a range of load vector angles and found that 10° gave the best agreement with the experimental data. This angle was also used and validated by others in a previous study which implemented the exact same *in vivo* loading experiment setup, with chukar partridges (Verner, 2017; Horasan et al., 2025). Distal contact surface nodes were constrained in translation to the point of load application, while rotation was allowed (i.e., pinned connection) to permit torsion at the midshaft which we observed to occur *in vivo* during background activities (Vitienes et al., 2023). Proximal contact surface nodes at the proximal end were held fixed in translation and rotation (i.e., fixed connection, or encastre).

**FIGURE 2:**
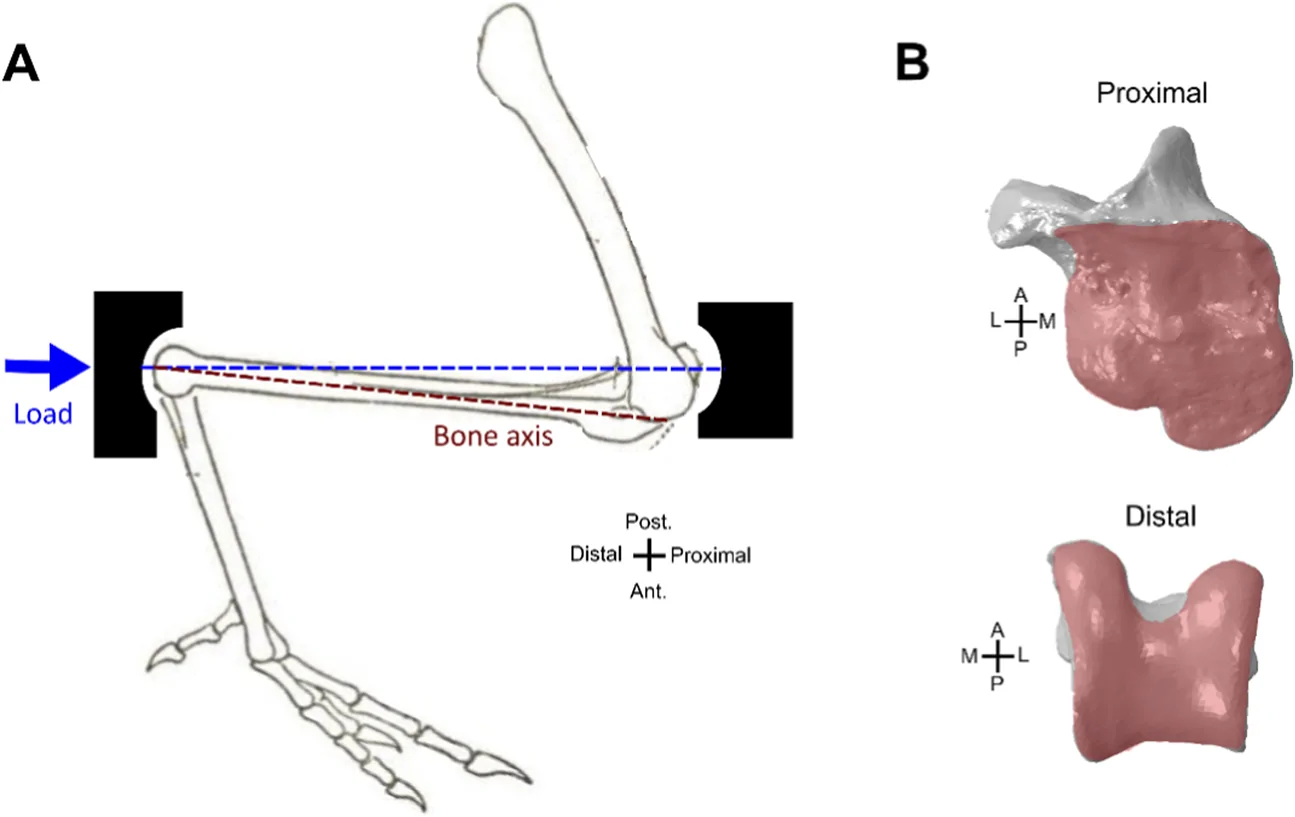
Finite element methods. **(A)** *In vivo* experimental loading setup simulated by the finite element modelling. Load is applied to the ankle in the direction of the load axis (dashed blue line) while the knee is held fixed in a flexed position. The load axis was rotated by approximately 10° in the posterior direction relative to the bone’s axis. **(B)** FEM knee (proximal) and ankle (distal) contact surfaces mimicking the *in vivo* experimental conditions.

#### 2.4.3 FEM material properties

We initially implemented a homogeneous material property approach where all tetrahedral mesh elements were assigned an elastic modulus of 11 GPa, based on experimental measurements at the femoral mid-diaphysis of age-matched female chickens (Wang et al., 2019). Differences in predicted strains and stresses across bones in the homogeneous models are a reflection solely of bone geometry as the elastic modulus and boundary conditions are the same. Subsequently, to include the effects of tissue mineral density heterogeneity on predicted strains and stresses in addition to bone geometry, each bone was modelled a second time with heterogeneous material properties where each tetrahedral mesh element was assigned a specific elastic modulus as a function of the tissue mineral density measured at that location in the microCT image (Razi et al., 2015).

##### 2.4.3.1 Derivation of density-elastic modulus relationship

Given the ash density ( $\rho_{ash}$ ) - elastic modulus (*E*) relationship (Razi et al., 2015).

$$E=k\;{\rho_{ash}}^{1.5}$$ (1)

where *k* is a constant, and assuming a linear relationship between ash density and attenuation coefficient μ defined by a constant *c,*

$$\rho_{ash}=c\;\mu$$ (2)

an attenuation coefficient - elastic modulus relationship can be derived as follows. Substituting $\rho_{ash}$ in Equation 1 by Equation 2,

$$E=k\;{\left(c\;\mu \right)}^{1.5}=\left(k\;c^{1.5}\right)\;\mu^{1.5}=k^{\prime }\;\mu^{1.5}$$ (3)

where $k^{\prime }=k\;c^{1.5}$ is a constant. Solving for *k’* in Equation 3, we obtain

$$k^{\prime }=\frac{E}{\mu^{1.5}}$$ (4)

Given that *k’* in Equation 4 is constant, for any two volumes of interest (VOI) *a* and *b*, with average attenuation coefficients ${\bar{\mu }}_{a}$ and ${\bar{\mu }}_{b}$ respectively,

$$k^{\prime }=\frac{E_{{VOI}_{a}}}{{{\bar{\mu }}_{a}}^{1.5}}=\frac{E_{{VOI}_{b}}}{{{\bar{\mu }}_{b}}^{1.5}}$$ (5)

Rearranging the second and third part of Equation 5, we obtain

$$E_{{VOI}_{a}}=\left(\frac{E_{{VOI}_{b}}}{{{\bar{\mu }}_{b}}^{1.5}}\right)\;{{\bar{\mu }}_{a}}^{1.5}$$ (6)

which denotes that, given a VOI_b_ of known elastic modulus $E_{{VOI}_{b}}$ and known average attenuation coefficient ${\bar{\mu }}_{b}$ , the elastic moduli of another VOI (here denoted by *a*) can be derived as a function of its average attenuation coefficient ${\bar{\mu }}_{a}$ .

Using imaging phantoms with microCT allows for the linear calibration of attenuation coefficients to units of tissue mineral density ( ρ ) (described above), and hence the variables of average attenuation coefficients in Equation 6 can be replaced by variables of average tissue mineral density:

$$E_{{VOI}_{a}}=\left(\frac{E_{{VOI}_{b}}}{{{\bar{\rho }}_{b}}^{1.5}}\right)\;{{\bar{\rho }}_{a}}^{1.5}$$ (7)

We thus used our measurements of the average tissue mineral density of the 6 FEA tibiotarsi (0.52 gHA/cm^3^, Figure 3) and reported mid-diaphyseal femoral tissue elastic moduli of age-matched female chickens, 11 GPa (Wang et al., 2019), as values for ${\bar{\rho }}_{b}$ and $E_{{VOI}_{b}}$ respectively in Equation 7 to derive a final Equation 8 relating our microCT-derived tissue mineral density to elastic modulus:

$$E=\left(\frac{11\;GPa}{{\left(0.52\;gHA/{cm}^{3}\right)}^{1.5}}\right)\;{{\bar{\rho }}_{a}}^{1.5}=\left(29.16\right){{\bar{\rho }}_{a}}^{1.5}$$ (8)

**FIGURE 3:**
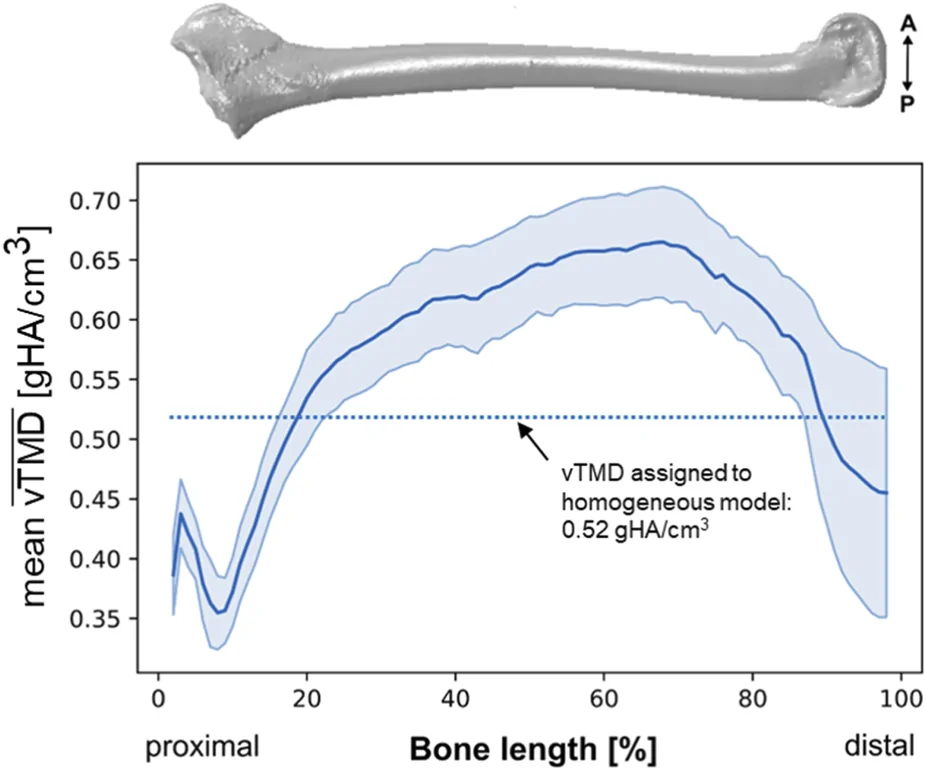
Mean average tissue mineral density along the tibiotarsi (n = 1/genetic strain [2]/housing [3]). For each bone, vTMD was averaged at 1% bone length intervals (denoted $\bar{\text{vTMD}}$ ), and the plotted mean ± sd is taken across the 6 bones included in the finite element analysis.

#### 2.4.4 FEM outcomes

Bone meshes were divided into 20 sections along the bone long axis, each section consisting of 5% of the bone length. Average principal strains (tensile: ${\bar{\varepsilon }}_{\max}$ , compressive: ${\bar{\varepsilon }}_{\min}$ ) and von Mises stress ( ${\bar{\sigma }}_{v}$ ) were calculated within each region without distinguishing cortical from trabecular bone. The von Mises stress is a measure of the overall mechanical response of the whole bone structure under loading, capturing the overall multiaxial stress state throughout the bone. Results are reported for each tibiotarsus ( $\bar{\varepsilon }$ and ${\bar{\sigma }}_{v}$ , values averaged within each 5% bone length section), or the mean across tibiotarsi (mean $\bar{\varepsilon }$ , mean ${\bar{\sigma }}_{v}$ ).

### 2.5 Bone structure

Thresholded whole bone images were aligned such that the bone’s longitudinal axis (defined as the chord joining the centroids at the proximal (5%) and distal (95%) ends of the bone) was in line with the global z-axis. At each slice, 2D measures of bone structure were computed and averaged across each 5% bone length section. We measured normalized bone curvature (C_R_, mm/mm), bone area (B.Ar, mm^2^), total area (T.Ar, mm^2^), bone area fraction (B.Ar/T.Ar, mm^2^/mm^2^), bone thickness (B.Th, mm), polar moment of inertia (pMOI = I_min_ + I_max_, mm^4^), eccentricity (I_min_/I_max_, mm^4^/mm^4^), normalized inner perimeter (I.Pm/T.Ar., mm/mm^2^), normalized outer perimeter (O.Pm/T.Ar, mm/mm^2^), normalized total perimeter (T.Pm/T.Ar, mm/mm^2^), and inner-to-outer perimeter ratio (I.Pm/O.Pm, mm/mm) (XamFlow, Lucid Concepts AG, Zürich, Switzerland). Curvature measurements were normalized to each bone’s midshaft anterior radius (distance between the midshaft slice centroid and the anterior periosteal surface), based on reported recommendations (Bertram and Biewener, 1992).

### 2.6 Data analysis

Differences between bones are reported as the difference in the given parameter in group A relative to group B ( ${\Delta \text{parameter}}_{A-B}=\left|A\right|-\left|B\right|,$ where A, B, and parameter are specified in each case). The correlation between bone morphological outcomes and predicted von Mises stress along the length of each bone was quantified by Pearson correlation coefficients, a normalized measure of covariance. For each bone, the Pearson’s correlation coefficient and p-value was measured for the set of pairs of average von Mises stress and average structural outcome within 10% bone length cross-sectional sections.

## 3 Results

### 3.1 FE modelled mechanical behaviour was not influenced by tissue mineral density heterogeneity

The inclusion of realistic, spatially heterogeneous vTMD in the heterogeneous models lead to negligible differences in modelled mechanical behaviour. Average volumetric tissue mineral density (vTMD, gHA/cm^3^) measured at 5% bone length sections ranged from 0.32 to 0.7 gHA/cm^3^ (Figure 3). The homogeneous FEMs were defined such that the bones had a fixed mineral density of 0.52gHA/cm^3^. At the proximal and distal regions, the homogeneous models had a mineral density up to 0.2 gHA/cm^3^ higher than the heterogeneous models (Figure 3). Generally, section-wise differences in heterogeneous and homogeneous model strains ranged from −222 to 201 με in tension and −376 to 267 με in compression. Peak mean average principal tensile strain (peak mean ${\bar{\varepsilon }}_{\max}$ ), compressive strain (peak mean ${\bar{\varepsilon }}_{\min}$ ), and von Mises stress (peak mean ${\bar{\sigma }}_{v}$ ) magnitudes from the heterogeneous models were numerically lower compared to the homogeneous models ( ${\Delta {\bar{\varepsilon }}_{\max}}_{\text{het}-\mathrm{hom}}$ = −126 με, ${\Delta {\bar{\varepsilon }}_{\min}}_{\text{het}-\mathrm{hom}}$ = −397 με, ${\Delta {\bar{\sigma }}_{v}}_{\text{het}-\mathrm{hom}}$ = −1 GPa) (Figures 4A,B,E,F). Both models exhibited similar proportions of tensile and compressive elements within each bone section (Figures 4C,D). Given the minimal differences in strain and stress magnitude and patterns along the length of the bone between heterogeneous and homogeneous models, subsequent results will be presented only for homogeneous models to isolate the effects of bone geometry on bone mechanics.

**FIGURE 4:**
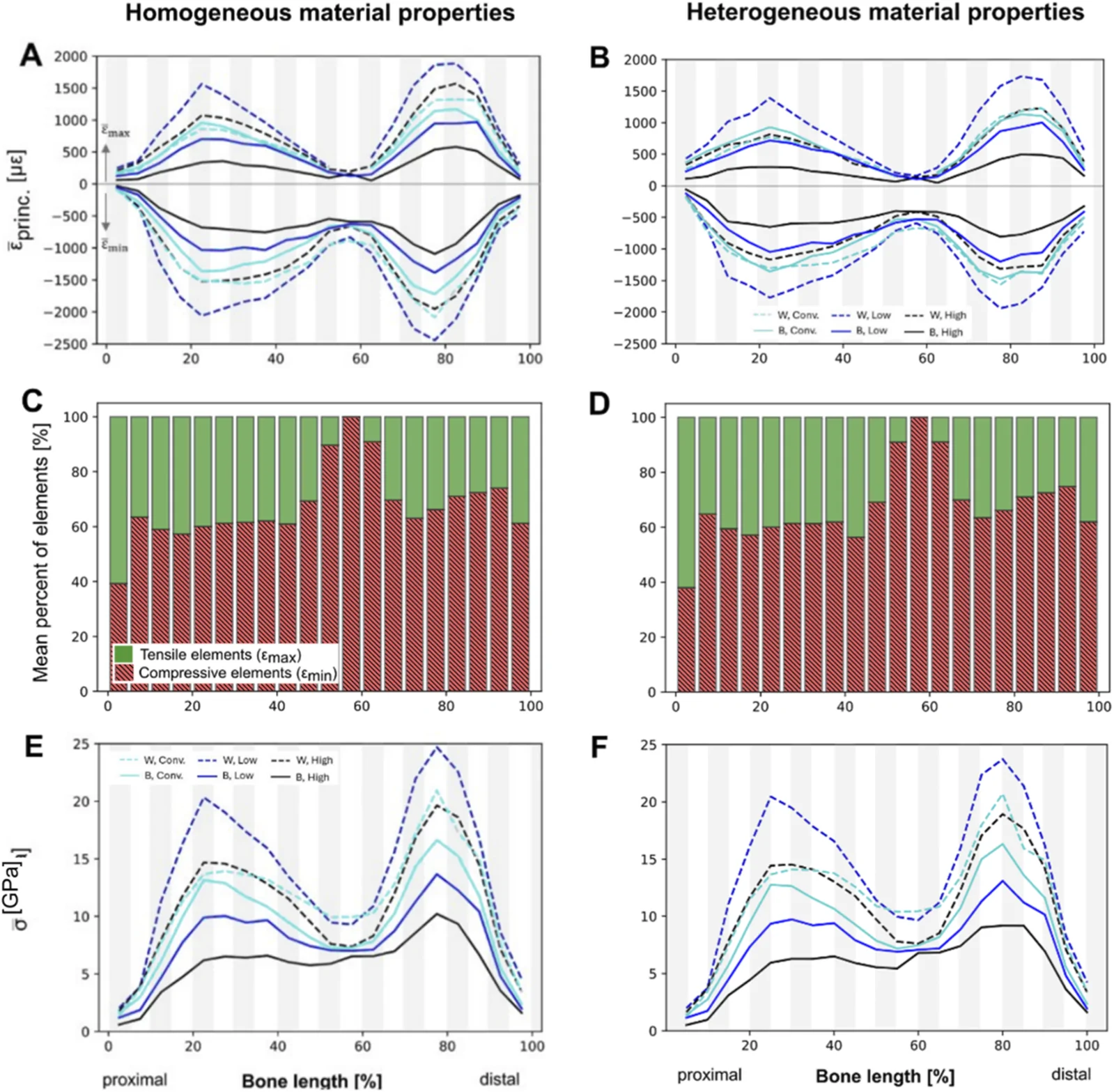
Estimated strains and stresses in each bone (n = 1/genetic strain [2]/housing [3]). Shaded regions on the backgrounds of plots A, B, E, F indicate the 5% bone length sections across which data from each element is averaged, for each bone. Results from homogeneous **(A,C,E)** and heterogeneous **(B,D,F)** models. **(A,B)** Average principal strain (tensile: ${\bar{\varepsilon }}_{\max}$ , compressive: ${\bar{\varepsilon }}_{\min}$ ) along the length of each bone. **(C,D)** Proportion of elements within each 5% bone length section which have a peak principal strain in tension (green) or compression (red, hashed), averaged across the 6 modelled bones. **(E,F)** Average von Mises stress ( ${\bar{\sigma }}_{v}$ ) along the length of each bone.

### 3.2 Structural determinants of bone mechanical behaviour

For each bone (N = 6) we quantified outcome measures of bone structure along the bone length and measured their correlation to modelled von Mises stresses along the bone length, to identify potential determinants of bone strength. All outcomes that we chose are generally thought to be associated with increased whole-bone stiffness, except for curvature which is generally associated to decreased stiffness. We found that bone area (B.Ar), total area (T.Ar), bone area ratio (B.Ar/T.Ar), bone thickness (B.Th), eccentricity (I_min_/I_max_), normalized outer perimeter (O.Pm/T.Ar), and inner-to-outer perimeter ratio (I.Pm/O.Pm) all significantly correlated (negatively) to von Mises stress–in other words, higher values in these outcomes was associated to lower stresses along the length of each bone - with mean Pearson correlation coefficients ranging from −0.46 to −0.64 (Figure 5).

**FIGURE 5:**
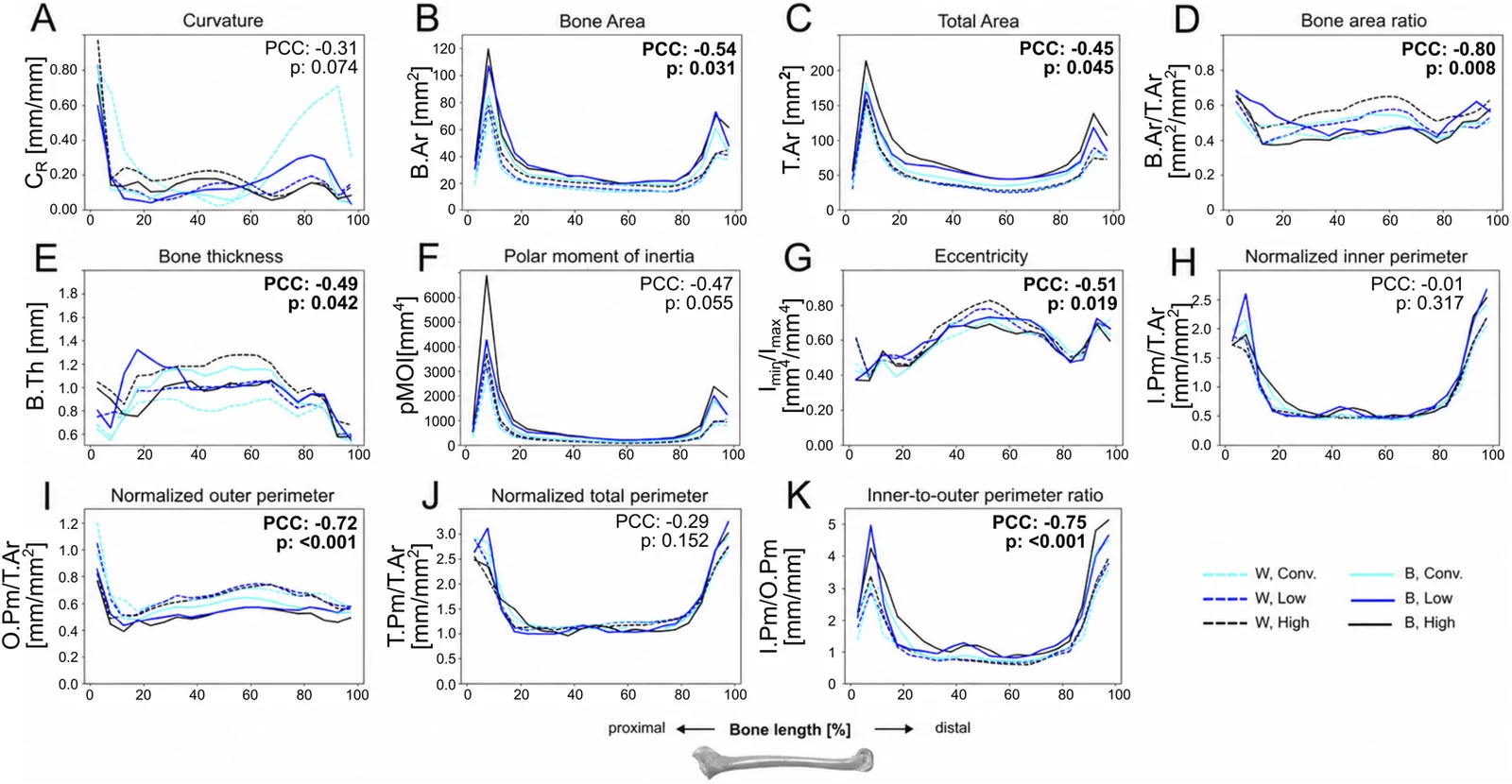
Correlation between structural measures and mechanical behaviour. **(A-K)** Plots of measures of each structural outcome, averaged across 5% bone length regions, for each bone. Each plot is annotated with the average Pearson correlation coefficient (PCC) and the median correlation p-value (p), for each bone, between the plotted outcome and simulated von Mises stress. Pairs are bolded when the p-value is significant.

### 3.3 Higher activity level was associated to lower mechanical stress in tibiotarsi from the brown- but not white-feathered genetic strain

We investigated how predicted strains and stresses differed between the tibiotarsi of sedentary (Conv) and active (Low and High) chickens. In all bones, the same pattern occurred in which stresses had local maxima at the proximal and distal regions; differences between the active and sedentary chickens’ tibiotarsi were most prominent in these distal regions (Figure 5). In brown-feathered chickens’ tibiotarsi we observed that the sedentary chicken (N = 1) had higher modelled strains compared to the active chickens (N = 2) (Figure 6). In white-feathered chickens’ tibiotarsi, there was not a consistent pattern in strains and stress between active and sedentary chickens (Figure 6).

**FIGURE 6:**
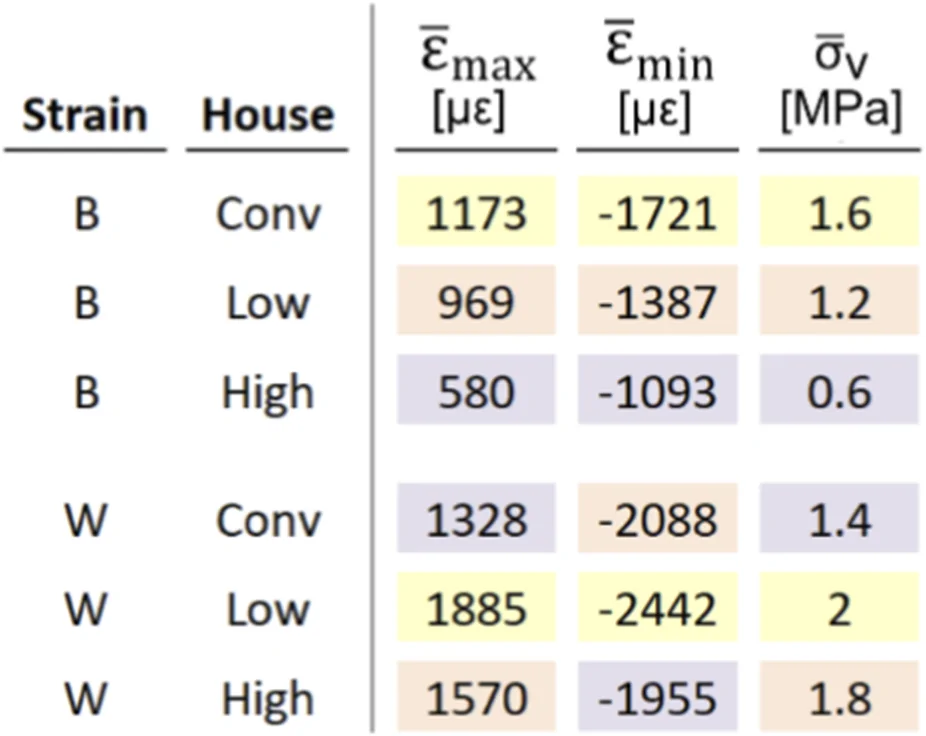
Peak principal strains ( ${\bar{\varepsilon }}_{\max},{\bar{\varepsilon }}_{\min}$ ) and von Mises stress ( ${\bar{\sigma }}_{v}$ ) for each bone included in our study. Colours highlight the highest (yellow), intermediate (orange), and lowest (purple) values for each genotype.

We identified a set of structural parameters that were correlated to decreases in von Mises stress in brown-feathered chickens’ tibiotarsi, which we considered as positive bone adaptations. These structural parameters, averaged across the whole bone, were compared between the active and sedentary brown-feathered chickens. Higher activity level was associated with higher bone area (B.Ar), total area (T.Ar), normalized inner perimeter (I.Pm/T.Ar), and inner-to-outer perimeter ratio (I.Pm/O.Pm) (Figure 7).

**FIGURE 7:**
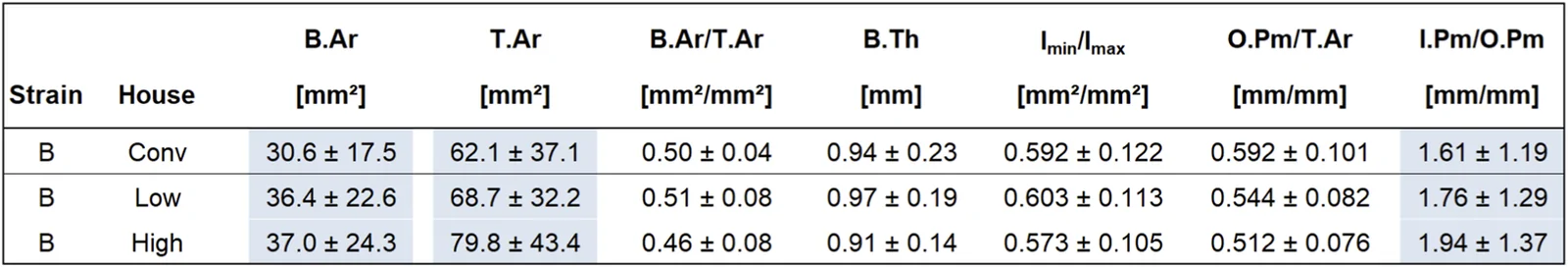
Structural determinants of mechanical behaviour. Mean (across the whole bone length) of the within-section average ±standard deviation of each the structural outcomes with significant correlation to von Mises stress (Figure 6), for the brown-feathered chickens’ bones modelled (n = 1/genetic strain (1)/housing (3)). Parameters which are lower in the sedentary chicken compared to the active chicken, and thus potential determinants of the simulated mechanical behaviour, are highlighted in blue.

Irrespective of loading history, we observed that the white-feathered chickens’ bones exhibited higher strain and stress magnitudes compared to the brown-feathered chickens’ bones (Figure 6).

### 3.4 FE predicted strains were validated by experimentally measured strains during controlled *in vivo* loading

The bone geometry was modelled by meshes which ranged from 2.49 to 3.66 x 10^6^ elements of an average characteristic length of 0.303 ± 0.028 mm (Table 1). Element characteristic length varied slightly across the six meshes (8% coefficient of variation), as a reflection of differences in bone microarchitecture. Mesh sensitivity analyses were previously conducted in a study modelling the chukar partridge tibiotarsus under near identical boundary conditions as ours, showing convergence at ca. 1.3 x 10^6^ mesh elements (Verner, 2017; Horasan et al., 2025). Experimentally measured strains differed from finite element predicted strains by on average 26 με in the homogeneous models and 118 με in the heterogeneous models (Table 1). These magnitudes amount to 5% and 24% respectively to the average strains measured during ground locomotion, in these same groups of chickens (Vitienes et al., 2023).

### 3.5 Tibiotarsi exhibited s-shaped deformation upon axial compression, with a 180deg neutral axis rotation at the midshaft

Principal tensile and compressive strain magnitudes, and average von Mises stress magnitude, all had similar distributions along the bone length, with local peaks in magnitude at the proximal and distal metaphyses, the latter being the global maximum (Figures 4A,B,E,F, 6A). At the proximal metaphysis, strain and stress magnitudes peaked at the 20%–25% bone length region (mean ${\bar{\varepsilon }}_{\max}$ = 916 μ&epsi;, mean ${\bar{\varepsilon }}_{\min}$ = −1,361 μ&epsi;, mean ${\bar{\sigma }}_{v}$ = 13 GPa). At the distal metaphysis, peak strains and stress magnitudes occurred at 75%–85% of the bone length (mean ${\bar{\varepsilon }}_{\max}$ = 1,248 μ&epsi;, mean ${\bar{\varepsilon }}_{\min}$ = −1781 μ&epsi;, mean ${\bar{\sigma }}_{v}$ = 18 GPa). The local strain and stress magnitude minima between these peaks occurred at 50%–60% bone length (mean ${\bar{\varepsilon }}_{\max}$ = 160 μ&epsi;, mean ${\bar{\varepsilon }}_{\min}$ = 708 μ&epsi;, mean ${\bar{\sigma }}_{v}$ = 8 GPa). At this location, the bone was completely under compression, whereas elsewhere there was a combination of tension and compression, with the proportion of tensile elements generally increasing with distance from this strain and stress magnitude local minimum towards the proximal and distal ends of the bone (Figures 4C,D). Global minima of strain and stress magnitude also occurred at a consistent location, namely, the 0%–5% bone length region (mean ${\bar{\varepsilon }}_{\max}$ = 163 μ&epsi;, mean ${\bar{\varepsilon }}_{\min}$ = 69 μ&epsi;, mean ${\bar{\sigma }}_{v}$ = 1 GPa) (Figures 4A,B,E,F). Along the bone length, stress varied across a range nearly as large as the peak stress magnitude achieved (94%). By observing strains plotted on the bone geometry, we saw that, from the anterior perspective, the bones exhibited convex bending (anterior tension, posterior compression) in the proximal region and concave bending (anterior compression, posterior tension) in the distal region (Figure 8A). This is to be expected given the bones’ natural s-shaped curvature present when not loaded (Figure 3). The transition in bending direction (rotation of the neutral axis by nearly 180°) occurred at the distal midshaft (52%–64% bone length) (Figure 8B), coinciding with the region of local strain and stress magnitude minima.

**FIGURE 8:**
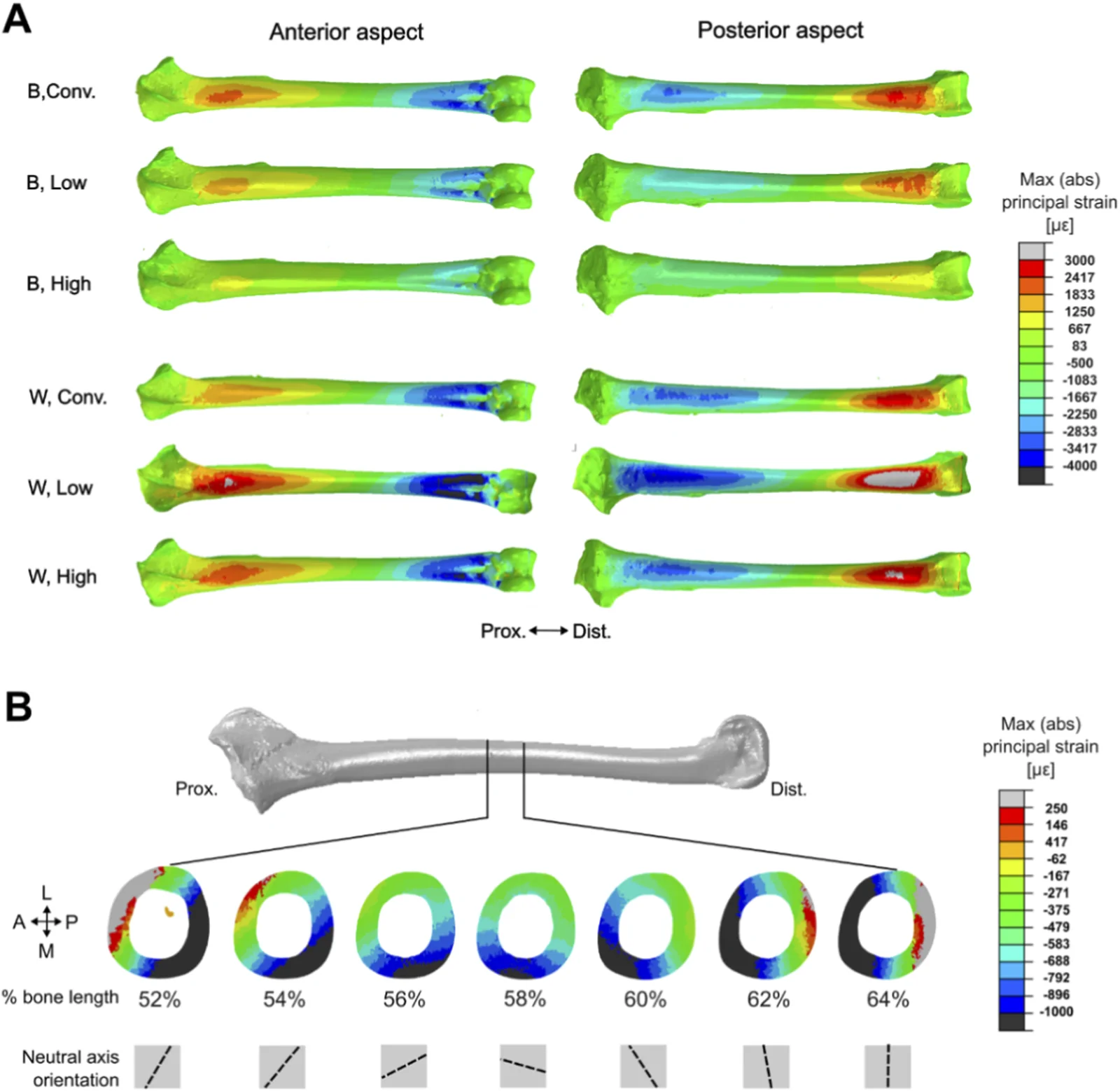
Principal strain distribution (n = 1 tibiotarsus/genetic strain [2]/housing [3]). For each element, the principal strain with the highest magnitude (max (abs)) is shown. Positive strains are tensile, negative strains are compressive. **(A)** Each bone seen from the anterior and posterior aspects. Bones are scaled to a common length. **(B)** Cross sectional strain distributions of an exemplary bone at the 52%–64% bone length region, where the transition from convex (proximal) to concave (distal) bending occurs (as visualized from the anterior aspect).

## 4 Discussion

The focus of this study was to investigate the structure-function relationship in young, female chicken tibiotarsi and identify bone stiffening adaptations to increased physical activity. We created finite element models to simulate experimental *in vivo* tibiotarsal loading and thereby estimate engendered principal strains and von Mises stress throughout entire tibiotarsi, which we validated with *in vivo* principal strain measurements at the anterior midshaft. We implemented the finite element models with either homogeneous or heterogeneous (tissue mineral density-derived) elastic moduli to assess the determinative effect of material properties on mechanical behaviour. With the homogeneous models, we assessed the effect of bone structure on the mechanical behaviour, by measuring the correlation between von Mises stress and a variety of structural parameters. We then compared patterns of mechanical behaviour and structural measures between active and sedentary chickens, in each genetic strain, to investigate by which adaptations can physical activity improve bone strength. We hypothesized that both tissue mineral density and structure would influence bone mechanical behaviour, and that chickens with more active loading histories would exhibit stiffer bones mainly attributed to adaptations in bone structure.

We found that consideration of tissue mineral density heterogeneity had negligible influence on modelled bone mechanical behaviour, and instead using the homogeneous models identified a set of structural parameters that did significantly correlated to von Mises stress in our sampled bones. Furthermore, we found that higher activity levels (low and high complexity aviary compared to conventional cages) were associated to lower modelled stresses in brown- but not white-feathered chickens’ tibiotarsi. This pattern coincided with increased (in active, brown-feathered chickens) bone area, total area, normalized inner perimeter, and inner-to-outer perimeter ratio.

### 4.1 Whole bone mechanical behaviour as a function of bone structure and material properties

To assess the influence of tissue mineral density heterogeneity on mechanical behaviour, we modelled each bone using both homogeneous elastic moduli and heterogeneous elastic moduli. The homogeneous models allowed us to isolate the effects of bone structure on the mechanical behaviour of the bone, and by comparing with the heterogeneous models we were able to assess whether the consideration of tissue mineral density heterogeneity in addition to bone structure plays a determining role in the bone’s mechanical behaviour. We observed substantial tissue mineral density heterogeneity in the bones: at the bone diaphyses, the tissue mineral density was up to 0.3 gHA/cm^3^ higher than at the metaphyseal regions. The elastic modulus assigned to the homogeneous models was derived as a function of the average tissue mineral density across the whole bone. Therefore, implementing a homogeneous material property approach not only removed the effect of mineral density heterogeneity, but also introduced a differential effect on the elastic modulus, dependent on the location in the bone where in some regions there was an increase in the elastic modulus in the homogenized model relative to the heterogeneous model (e.g., metaphyses, endosteal surface), and in other regions a decrease (e.g., diaphysis, intracortical region).

There was a negligible difference in engendered stress magnitudes between the heterogeneous and homogeneous models, indicating that changes in tissue mineral density at or below 0.2 gHA/cm^3^, which corresponds to approximately 25% of the average cortical diaphyseal vTMD of age- and genetic strain-matched chickens (Vitienes et al., 2026), were not influential on engendered von Mises stress levels. We did however observe higher peak principal strain in the homogeneous compared to the heterogeneous models, with average peak strains differing by up to 397 με; in other words, the homogeneous model is more conservative. This difference is likely physiologically relevant, since we have previously shown in chickens from the same genotype and housing groups, that *in vivo* locomotor peak principal strains range from 525 to 1,016 με in magnitude (Vitienes et al., 2023). Given that the von Mises stress is purely a function of the three maximum shear stresses (stress deviator tensor), the fact that principal strains but not von Mises stress differed between homogeneous and heterogeneous models suggests that tissue mineral density heterogeneity and variations in magnitude of up to 0.2gHA/cm^3^ did not contribute to the bones’ ability to resist shear strain, but was relevant for resisting normal principal strain. These results also suggest that future studies performing FEA on avian tibiotarsi can directly implement a homogeneous material property model if the main outcome of interest is von Mises stress, without the need to create the much more complex heterogeneous FE models.

We next focused on the homogeneous models to seeking structural outcomes that correlate to our modeled von Mises stress. Most outcomes measured (bone area, total area, bone area ratio, bone thickness, normalized inner perimeter, and inner-to-outer perimeter ratio) significantly, and negatively, correlated to stress along the length of each bone, and are therefore potential candidate structural measures that can be used as indicators of whole-bone stiffness, and selected for during breeding.

### 4.2 Differences in bone mechanical behaviour between active and sedentary chickens

We observed a robust pattern in which both principal strains and von Mises stress had a bimodal distribution along the bone length, peaking at the proximal and distal metaphyses. It is in these regions where we saw most notable differences between genetic strains and loading history groups. In looking at differences in mechanical strains and stress between active and sedentary bones, we hypothesized that chickens with more experience of physical activity would have acquired bone-stiffening adaptations such that, for a given applied force, their bones would deform less compared to chickens with a more sedentary history. In a previous study we quantified, at the flock level, activity levels of the groups of chickens reared in aviaries from which those included in this current study were sampled. Aerial locomotion, defined as jumping to ascend or descend from perches or platforms with or without wing-flapping, was observed as early as 1 week of age in both genetic strains and increased over time as the birds grew and used various structures in the aviary. By design, these activities were impossible to perform in conventional cages. Therefore, we expected to see differences in mechanical behaviour between aviary- and cage-reared chickens of both genetic strains resulting from adaptations to different loading histories.

Instead, we observed that only in the brown-feathered genetic strain did the Conv chicken’s tibiotarsus exhibit higher strains and stress than the active (Low and High complexity aviary) chickens. In the brown-feathered genetic strain we then compared patterns of whole-bone average structural outcome measures with peak stress levels across the sampled bones of the active and sedentary chickens, with the expectation that bones with higher stress would have lower values in stiffening outcomes. We found this to be the case for bone area, total area, normalized inner perimeter, inner-to-outer perimeter ratio. With increased accessibility to computed tomography in the poultry industry, these results provide outcome measures that can be used as surrogate measures for bone stiffness, and which can be used for selective breeding.

Comparing genotypes, most notably we saw that the tibiotarsi of brown-feathered chickens were better able at resisting deformation when subjected to our simulated *in vivo* controlled loading conditions. The brown-feathered Lohmann Brown genetic strain has a higher average body weight compared to the Lohmann Selected Leghorn Lite (Vitienes et al., 2023) and larger muscles (Pufall et al., 2021). Therefore, their bones are adapted to supporting these higher load levels habitually, which likely explains this finding. We previously quantified bone microstructural properties at the tibiotarsus midshaft of chickens in these groups (Vitienes et al., 2026) and found that although white-feathered bones had higher cortical thickness and cortical area fraction, they had lower polar moment of inertia and robusticity. Together these data suggest that the polar moment of inertia and robusticity adaptations are more successful in resisting bone deformation, at least at the tibiotarsus midshaft when subjected to our controlled loading conditions.

### 4.3 General mechanical behaviour of the tibiotarsus under controlled axial compression

The fact that both principal tensile and compressive strains peak simultaneously with the peak compressive strain being slightly higher than the peak tensile, indicates that there is a combination of compression and bending in these regions (Main, 2021), as expected in such a loading scenario. The heat maps in Figure 8 show anterior convex and concave bending in the proximal and distal ends, respectively. Bending occurs when the axis of applied load does not intersect with the centroid of the bone in the plane that is perpendicular to said load. This scenario occurs during off axis loading, or when there is a moment arm due to curvature of the bone. In the case of our FE models, load was applied along an axis rotated 10° posteriorly relative to the bone’s axis, therefore producing a moment arm along all bone cross-sections. It has been theorized that, although bone is weaker in bending compared to pure compression, structural and tissue-level adaptations that enable bending of the bone are evolutionarily favourable, as they result in a predictable direction of deformation under variable load directions (Bertram and Biewener, 1988).

Chickens naturally perform a variety of activity types such as ground and ramp locomotion, wing-assisted locomotion, jumping, and perching (Pufall et al., 2021; Rentsch et al., 2023), all of which likely produce variable magnitudes and directions of ground reaction forces acting on the tibiotarsi; this scenario would support the need to have structural features that result in consistent bone deformation directions under these variable conditions. These results also highlight how different sites of the bone undergo different modes of loading (e.g., mainly axial compression and shear at the midshaft compared to substantial bending at the metaphyses) and therefore serve to inform mechanical testing protocols that aim to replicate physiological failure conditions.

## 5 Conclusion

In this current study we present for the first time finite element modelling of the chicken tibiotarsus. Overall our results on the general mechanical behaviour of the tibiotarsus agree with the single other study that has performed finite element modelling of *in vivo* controlled loading of an avian tibiotarsus, specifically the chukar partridge (Verner, 2017; Horasan et al., 2025), which modelled the same *in vivo* loading conditions as we did. They showed that peak strains occur at the proximal and distal metaphyses, whereas at the midshaft strains were lower. Our von Mises stress data suggests that there is substantial shear occurring in addition to bending and compression. In the chukar partridge they similarly show that the tibiotarsus undergoes a combination of bending, compression, and torsion-induced shear. In the chukar partridge, however, the highest strains occur at the proximal metaphysis as opposed to the distal metaphysis in the chicken, and minimum strains occur relatively more proximal to that what we saw in the chicken tibiotarsus. Differences in modelled mechanical behavior between the two avian species are expected within the load predictability hypothesis, due to their differences in the size (chukar: 0.4 kg, chicken: 1.3 kg). In small birds like the chukar partridge, the proportionally predominant source of load is from muscle contraction, as opposed to ground reaction forces, which produce consistent localized forces to the sites of muscle attachment (Biewener, 1989). Chickens alternatively, being nearly three times heavier than the chukar, experience proportionally exponentially higher ground reaction forces which are more variable in magnitude and direction. In the context of the load predictability hypothesis, we would expect a higher need for structural adaptations that control the strain mode distribution in the chickens compared to the chukar; in other words, bone structure in the chukar and chicken would be tailored to distinct needs, which in turn may explain why under the nearly identical boundary conditions of our finite element models, we see different stress patterns, specifically sites of peak stress, in the chicken and chukar.

Our study has limitations. Due to the extensive time required to create and run each model, and we performed finite element analyses on a sample size of n = 1, and therefore were restricted to evaluating numerical patterns without statistical analyses. We validated our finite-element models with strains measured during *in vivo* controlled loading at the midshaft, and limitations did not allow us to experimentally measure strains at other, distal regions, *in vivo* or *ex vivo*. However, our findings regarding the patterns of predicted strain along the bone length agree with finite-element predictions of strain due to axial compressive controlled loading in chukar partridges with similar whole-bone morphology (Verner, 2017; Horasan et al., 2025) discussed above. Future investigations involving *in vitro* strain gauging of multiple regions along the length of the bones are needed to confirm the finite element predicted strains. In terms of experimental design, our ability to interpret our strain and stress findings in relation to the differences in activity levels between aviary styles is limited by our ability to quantify the physical activity levels of individual chickens. The common practice in poultry research is to identify focal birds and observe them for a given amount of time (e.g., 5 min) and record the occurrence of different activity types (Pufall et al., 2021; Rentsch et al., 2023). As technologies advance, it may be feasible in the future to follow individual birds for extended periods of time and collect additional data such as ground reaction forces of the chickens in the different housing systems, which would aid in the interpretations of our current findings. We could however assume differences in activity levels between the caged and aviary groups because cages do not allow for any jumping and aviaries require that each chicken jump between levels to reach food, water, and litter.

A common challenge with finite element modelling, is to determine the necessary level of complexity to achieve sufficiently accurate measurements. Our finding that the homogeneous and heterogeneous models had negligible differences in modelled von Mises stress, magnitudes and patterns throughout the bone, justify the use of computationally efficient homogeneous models in similar comparative contexts. This is a particularly valuable finding given the growing use of computed tomography and CT data-derived analyses in commercial poultry settings for selective breeding. This results also agrees with recent findings in humans that indicate that bone biomechanics and fracture resistance is largely determined by bone structure, and that fracture risk is better assessed by structural parameters than bone mineral density.

Our findings also have implications in the broader field of bone adaptation and comparative biomechanics. Studies in humans have also shown that while modelled strain outcomes may be more sensitive to material properties, stress magnitudes and patterns are almost entirely governed by bone structure, in long bones (Helgason et al., 2008; Chytła and Wojnarowska, 2025). This dominance of structure over tissue mineral density heterogeneity is in agreements with principles of allometry in skeletal biology–bone material properties are relatively constant across diverse body sizes whereas skeletons scale in structure according to variations in body mass (and therefore, mechanical stresses in the bones). This relationship however should not be generalized to all bones–in human vertebrae, material heterogeneity is important and local density variations have been shown to dictate where failure may occur (Maquer et al., 2014).

In summary, our key findings were: 1) Variations in tissue mineral density of up to 0.2 gHA/cm^3^ were not influential on engendered stress magnitudes or patterns along the bone; 2) Bone area, total area, bone area ratio, bone thickness, eccentricity, normalized outer perimeter, and inner-to-outer perimeter ratio were robust negative correlates of bone stress; 3) Increased history of physical activity was associated with stiffer bones in the Lohmann Brown, but not Lohmann LSL Lite genotype of female chickens and the lower stresses in active compared to sedentary Lohmann Brown chickens was associated with higher bone area, total area, normalized inner perimeter, and inner-to-outer perimeter ratio. Future research is needed to assess the effects of other determinants of whole-bone stiffness and strength such as porosity, osteon density, collagen fiber orientation, osteocyte lacunocanalicular network topology, and microdamage accumulation. These data inform poultry housing practices, as well as mechanical testing protocols that aim to replicate *in vivo* loading. Importantly, beyond bone mineral density, these data identify structural outcomes as candidates for genetic selection, to target improved bone strength and reduce fracture risk in egg laying hens.

## Data Availability

Data included in this study can be found here: https://github.com/vitienesIsa/Pullet_FE.
